# Replication-Competent Influenza A and B Viruses Expressing a Fluorescent Dynamic Timer Protein for *In Vitro* and *In Vivo* Studies

**DOI:** 10.1371/journal.pone.0147723

**Published:** 2016-01-25

**Authors:** Michael Breen, Aitor Nogales, Steven F. Baker, Daniel R. Perez, Luis Martínez-Sobrido

**Affiliations:** 1 Department of Microbiology and Immunology, University of Rochester School of Medicine and Dentistry, 601 Elmwood Avenue, Rochester, New York, 14642, United States of America; 2 Department of Population Health, University of Georgia, 953 College Station Road, Athens, Georgia, 30602, United States of America; Mount Sinai School of Medicine, UNITED STATES

## Abstract

Influenza A and B viruses (IAV and IBV, respectively) cause annual seasonal human respiratory disease epidemics. In addition, IAVs have been implicated in occasional pandemics with inordinate health and economic consequences. Studying influenza viruses *in vitro* or *in vivo* requires the use of laborious secondary methodologies to identify infected cells. To circumvent this requirement, replication-competent infectious influenza viruses expressing an easily traceable fluorescent reporter protein can be used. Timer is a fluorescent protein that undergoes a time-dependent color emission conversion from green to red. The rate of spectral change is independent of Timer protein concentration and can be used to chronologically measure the duration of its expression. Here, we describe the generation of replication-competent IAV and IBV where the viral non-structural protein 1 (NS1) was fused to the fluorescent dynamic Timer protein. Timer-expressing IAV and IBV displayed similar plaque phenotypes and growth kinetics to wild-type viruses in tissue culture. Within infected cells, Timer’s spectral shift can be used to measure the rate and cell-to-cell spread of infection using fluorescent microscopy, plate readers, or flow cytometry. The progression of Timer-expressing IAV infection was also evaluated in a mouse model, demonstrating the feasibility to characterize IAV cell-to-cell infections *in vivo*. By providing the ability to chronologically track viral spread, Timer-expressing influenza viruses are an excellent option to evaluate the *in vitro* and *in vivo* dynamics of viral infection.

## Introduction

IAV and IBV infections are an important cause of human deaths in the United States (US) with approximately 3,700 fatalities in 2013 [1] and upwards of 500,000 worldwide [2]. In addition, pandemics caused by IAV are well documented. In the 20^th^ century, three IAV pandemics occurred with the most devastating one in 1918, known as Spanish flu, that killed between 30–50 million people [3]. April 2009 marked the emergence of an H1N1 IAV responsible for the first pandemic of the 21^st^ century. It has been estimated that the 2009 pandemic H1N1 IAV infected over 60 million people resulting in approximately 275,000 hospitalizations and 12,000 deaths in the US alone [4]. Globally, it is estimated that an excess of 200,000 deaths occurred from influenza and secondary complications during this pandemic [5]. Although perceived as less dangerous, IBV infections are substantial contributors to pediatric deaths. In 2011, 38% of all influenza-related childhood fatalities in the US were due to IBVs [6].

IAV and IBV belong to the family *Orthomyxoviridae*, having in common host-derived lipid envelopes and segmented RNA genomes of negative polarity [7]. IAV and IBV contain 8 genomic viral RNA (vRNA) segments in the form of viral ribonucleoprotein particles (vRNPs) coated with multiple copies of the nucleoprotein (NP) and associated to the tripartite RNA dependent RNA polymerase complex (PB2, PB1 and PA) [7, 8]. vRNPs constitute the transcription/replication competent units of influenza viruses and as such are incorporated into progeny particles.

The development of plasmid-based reverse genetics to generate recombinant influenza viruses [9, 10] has proven to be essential for developing influenza vaccines [11–13] and making strides in understanding the biology of these important human pathogens [14–16]. More recently, this technology has allowed for the generation of replication-competent influenza viruses expressing reporter genes, as novel powerful tools to track viral infections both *in vitro* and *in vivo* [13, 17–32].

Currently, several replication-competent IAVs have been described that express static fluorescent or luminescent proteins through modification of the non-structural (NS) gene segment 8 [13, 17, 20, 21, 23–25, 33, 34]. IAV and IBV NS segments encode both the non-structural protein 1 (NS1) as a linear transcript and the nuclear export protein (NEP), via an alternative mRNA splicing mechanism [35]. NS1 coordinates viral antagonism of the antiviral host response through interferon (IFN) inhibition [35, 36], and NEP is required to export vRNPs from the nucleus to budding virions [7]. NS1 has often been utilized for reporter gene expression because of its high copy number in infected cells and short nucleotide length [37]. Previously generated reporter-expressing viruses allow for infection to be observed *in vitro* and *in vivo*. However, such studies have not allowed determining the origin or chronology of infection. Thus, systems that can be used to track influenza infections both spatially and temporally are highly beneficial. A dynamic fluorescent protein Timer was developed that changes its emission spectra from green to red over time and could allow for tracking influenza infections in more detail [38]. Timer is derived from the Discosoma Red (DsRed) fluorescent protein and contains two point mutations that confer a higher quantum yield and the spectral shift phenotype [38].

We describe the generation of replication-competent IAV and IBV expressing Timer (IAV-Timer and IBV-Timer, respectively) fused to the viral protein NS1. *In vitro*, Timer-expressing IAV and IBV have similar growth kinetics compared to their respective wild-type (WT) counterparts. Using multiple approaches, including fluorescent microscopy and plaque assays, we were able to differentiate primary from secondary infected cells. Timer expression and spectral shift was quantified in infected cells using a fluorescence plate reader and flow cytometry. Importantly, IAV-Timer was useful to evaluate the dynamics of viral infections in mouse lungs using an *in vivo* imaging system (IVIS). These studies constitute proof-of-principle of the usefulness for recombinant IAV- and IBV-Timer viruses to study viral infection dynamics.

## Material and Methods

### Cells

Human embryonic kidney 293T (ATCC CRL-11268) and Madin-Darby canine kidney (MDCK, ATCC CCL-34) cells were maintained in Dulbecco’s modified Eagle’s Medium (DMEM, Mediatech, Inc.) containing 10% fetal bovine serum (FBS, Atlanta biological) and 1% PSG (penicillin, 100 units/mL; streptomycin, 100 μg/mL; L-glutamine, 2mM; Mediatech, Inc.) at 37°C in 5% CO_2_. After viral infections, cells were maintained at 33°C in a 5% CO_2_ atmosphere in DMEM containing 0.3% bovine serum albumin (BSA), 1% PSG, and 1 μg/ml tosylsulfonyl phenylalanyl chloromethyl ketone (TPCK)-treated trypsin (Sigma).

### Timer constructs and influenza virus rescues

To rescue Timer-expressing IAV and IBV, the open reading frame (ORF) of Timer protein (Clontech) was fused to the NS1 of IAV (A/Puerto Rico/8/1934 H1N1) [13, 21] or IBV (B/Brisbane/60/2008) as previously described [13, 21]. Briefly, the NS segment was modified such that the NS1-Timer fusion sequence was followed by the porcine teschovirus-1 (PTV-1) 2A autoproteolytic cleavage site followed by NEP [13]. Standard cloning methods were used to insert the modified NS gene segments into plasmids pDZ [39] and pDP-2002 [33] for IAV and IBV rescue transfections, respectively. Plasmid constructs were confirmed by sequencing (ACGT, Inc.).

IAV-Timer was rescued in the A/California/04_NYCIE_E3/2009 (pH1N1) virus backbone [40]. Virus rescues were performed as previously described [11, 33]. Briefly, eight ambisense plasmids containing a genomic viral segment (PB2, PB1, PA, HA, NP, NA, M, and NS WT or NS-Timer) of either IAV or IBV were co-transfected into a co-culture of 293T and MDCK cells using Lipofectamine-2000 (Invitrogen). At 48–72 hours post-transfection, tissue culture supernatants (TCS) were collected, clarified and used to infect fresh MDCK cells. All viruses were plaque purified and scaled up in MDCK cells. Viral titers were determined by plaque assay [13].

### Viral plaque assays

Confluent monolayers of MDCK cells in 6-well plates (10^6^ cells/well) were infected with 10-fold serial dilutions of Timer-expressing IAV or IBV. Infected cells were incubated at 33°C and at 3–4 days post infection (dpi), cells were fixed with 2.5% paraformaldehyde in phosphate buffered saline (PBS) and the agar overlays were carefully removed and replaced with PBS. For the bull’s eye assay, a plaque assay where fluorescence expression is evaluated under a fluorescent microscope, green and red fluorescent images of individual plaques were taken using a fluorescence microscope (Olympus IX81) and camera (QIMAGING, Retiga 2000R), and merged using Photoshop CS4 (Adobe). For plaque images and immunostaining, green and red fluorescent IAV or IBV foci were imaged using a Kodak image station (4000MM Pro molecular imaging system; Carestream Health, Inc., NY) and Kodak molecular software (v5.0.1.30). Foci were then immunostained as previously described [13] using specific NP monoclonal antibodies (MAb) for IAV (HT-103) [41] or IBV (AbCam B017) and vector kits (Vectastain ABC kit, DAB HRP Substrate Kit; Vector) following the manufacturer’s conditions. IAV and IBV WT were used as plaque controls.

### Multicycle replication kinetics

MDCK cells (5x10^5^ cells/well, 12-well plate format, triplicates) were infected at a multiplicity of infection (MOI) of 0.001 and at the designated times post-infection, TCS were collected and viral titers determined by immunofocus assay (focus forming units per ml, FFU/ml) as previously described [13], using IAV (HT-103) [41] or IBV (AbCam B017) NP specific MAbs.

### Protein gel electrophoresis and Western blot analysis

MDCK cells were mock infected or infected (MOI of 3) and harvested at 24 hours post-infection (hpi). Cells were lysed in passive lysis buffer (Promega), centrifuged for 20 minutes at 14,000 rpm, and the cell lysates were frozen at -80°C until use. Proteins from lysates were separated using 10% SDS-PAGE, transferred to a nitrocellulose membrane, blocked in 5% fat-free powdered milk dissolved in PBS containing 0.1% Tween-20 (PBS-T) and incubated overnight at 4°C with specific MAb or polyclonal (PAb) antibodies against IAV [39] or IBV [42] NS1 proteins; IAV (MAb BEI NR-4282) or IBV (MAb AB017, AbCam) NP; and β-actin (MAb A3854, Sigma). Antibody-bound membranes were then incubated with species-specific secondary horseradish peroxidase (HRP)-conjugated antibodies (GE Healthcare UK) and protein bands were detected by chemiluminescence (HyGlo, Dennville Scientific Inc.) on a Kodak image station (4000R).

### Fluorescence plate reader assays

Confluent MDCK cells (4x10^4^ cells/well, 96-well plate format, triplicates) were infected with either IAV- or IBV-Timer at low (0.001) or high (3) MOI. For images, infected cells at the indicated time points (considering 0 hpi after 1 hour of adsorption onto cells) were fixed with 4% paraformaldehyde. To quantify fluorescence expression, cell monolayers were washed with PBS, and green and red fluorescence expression was measured using a fluorescence microplate reader (DTX 880, Beckman Coulter). Fluorescence values were calculated by subtracting the background signal of mock-infected cells from each time point’s mean and normalized by setting 100% infection as time point at the higher value for both viruses and fluorescence spectrums.

### Flow Cytometry

MDCK cell monolayers (10^6^ cells/well, 6-well plate format, triplicates) were infected at low (0.001) or high (3) MOI with IAV-Timer. Cells infected with PR8 GFP [21] and PR8 DsRed (unpublished) were used as green and red fluorescent virus controls, respectively. At the indicated times post-infection (considering 0 hpi after 1 hour of adsorption onto cells), cells were collected and resuspended in 1 mL of PBS containing 5% fetal bovine serum (FBS), pelleted by centrifugation, and fixed in suspension with 4% paraformaldehyde in PBS for 20 minutes at room temperature. Cells were then washed twice PBS/5% FBS and stored at 4°C until analysis with a C6 four-color flow cytometer (Accuri). Data were analysed using FlowJo software (Tree Star), and gates were set based on mock-infected cells.

### Genetic stability of reporter Timer-expressing viruses in cell culture

To determine the stability of the Timer-expressing IAV and IBV, MDCK cells were infected (MOI of 0.01) as previously describe [25]. After 5 subsequent serial passages, plaque assays were performed to evaluate Timer fluorescent-expressing plaques. The presence of red and green fluorescent plaques (out of 40 to 50 counted plaques) was analyzed by fluorescent microscopy as indicated above.

### Mouse studies

Female 6-to-8-week-old C57BL/6 mice were maintained in the animal care facility at the University of Rochester under specific pathogen-free conditions. All animal protocols were performed in accordance and approved by the University of Rochester Committee of Animal Resources. During the course of the study, no mice experienced an unintended death or were humanely euthanized due to excessive illness. Mice were anesthetized intraperitoneally with 2,2,2-tribromoethanol (Avertin) and then inoculated intranasally (i.n.) with PBS or 10^5^ plaque-forming units (PFU) of IAV-Timer and monitored daily for signs of clinical disease. At 24, 48, 72 and 96 hpi, mice were euthanized by administration of a lethal dose of Avertin and exsanguination. Expression of Timer in whole excised lungs was analyzed by evaluating GFP and DsRed fluorescence using an IVIS Spectrum multispectral imaging instrument (Caliper Life Sciences, Inc.). The images were acquired and analyzed with the LivingImage 3.0 software to determine radiant efficiency [p/s/cm2/sr] / [mW/cm2]. Induction of fluorescence signal was normalized to mock-infected animals. To evaluate viral replication, mouse lungs were homogenized and virus titers determined using an immunofocus assay (FFU/ml) as indicated above.

### Statistical analysis

Mean values, standard deviations, and statistical analysis using a two-tailed, student’s t-test, were calculated using Microsoft Excel software.

## Results

### Generation and characterization of recombinant IAV and IBV expressing the fluorescent dynamic Timer protein (IAV- and IBV-Timer)

**Fig 1:**
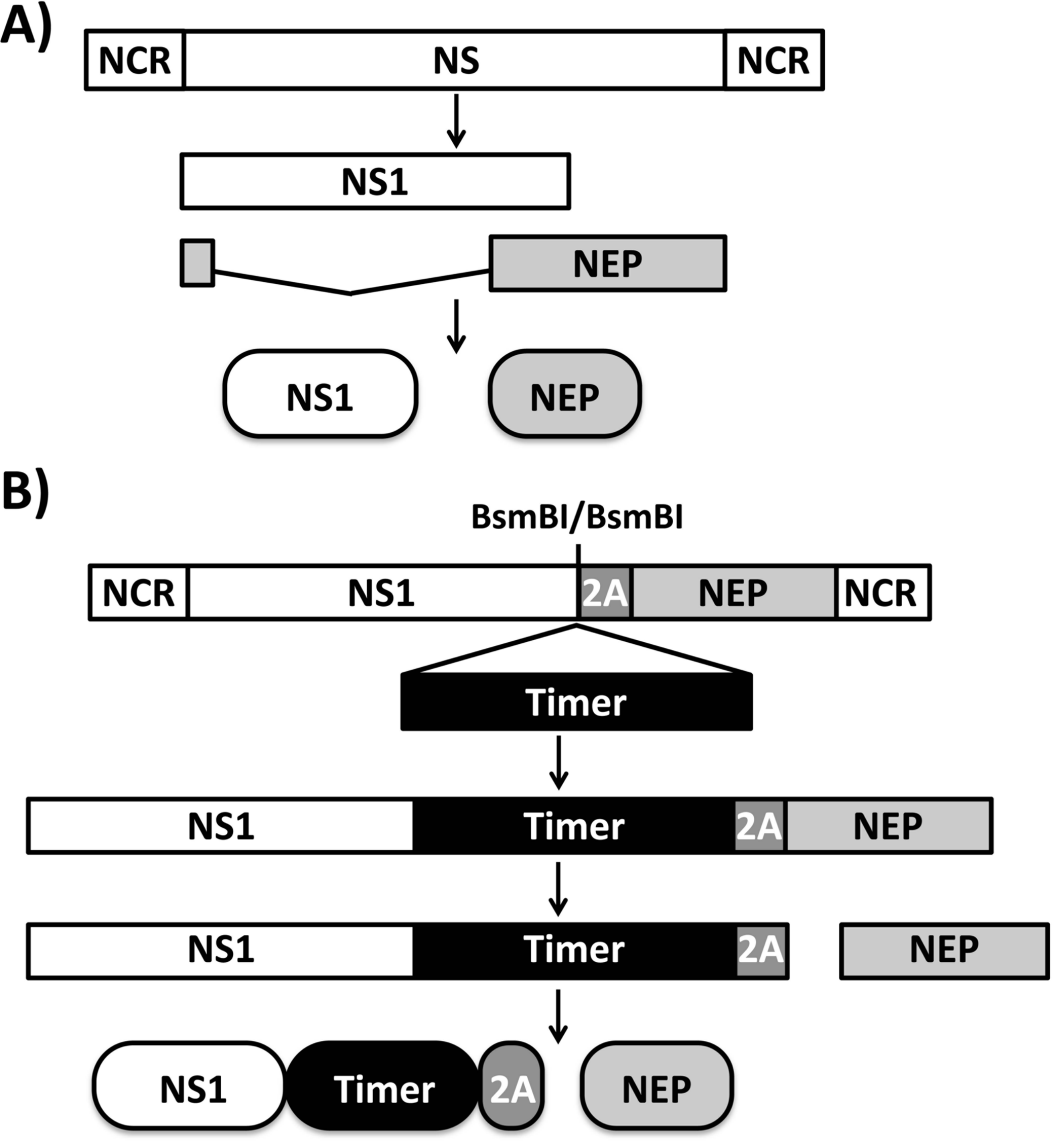
Schematic representation of wild-type (A) and Timer-expressing (B) influenza A and B NS segments. Influenza NS segment viral products are indicated by white (NS1) or gray (NEP) boxes. Timer fluorescent protein and the porcine teschovirus-1 (PTV-1) 2A site are indicated by black and gray boxes, respectively. NCR, non-coding regions. BsmBI restriction sites used to clone Timer between NS1 and PTV-1 2A are indicated.

To generate replication-competent influenza viruses expressing a reporter protein, the presence of the fluorescent protein or the gene cannot disrupt viral replication or packaging. It has been previously shown that a replication-competent IAV expressing a fluorescent protein can be generated by fusing the reporter to NS1 [13, 17, 21–25]. Thus, the fluorescent Timer ORF was fused to that the C-terminus of NS1 in both IAV and IBV NS segments (Fig 1A). The strategy allows for collinear expression of NS1-Timer and NEP (Fig 1B) in the background of recombinant IAV (A/California/04_NYCIE_E3/2009, pH1N1) and IBV (B/Brisbane/60/2008) Timer-expressing viruses.

**Fig 2:**
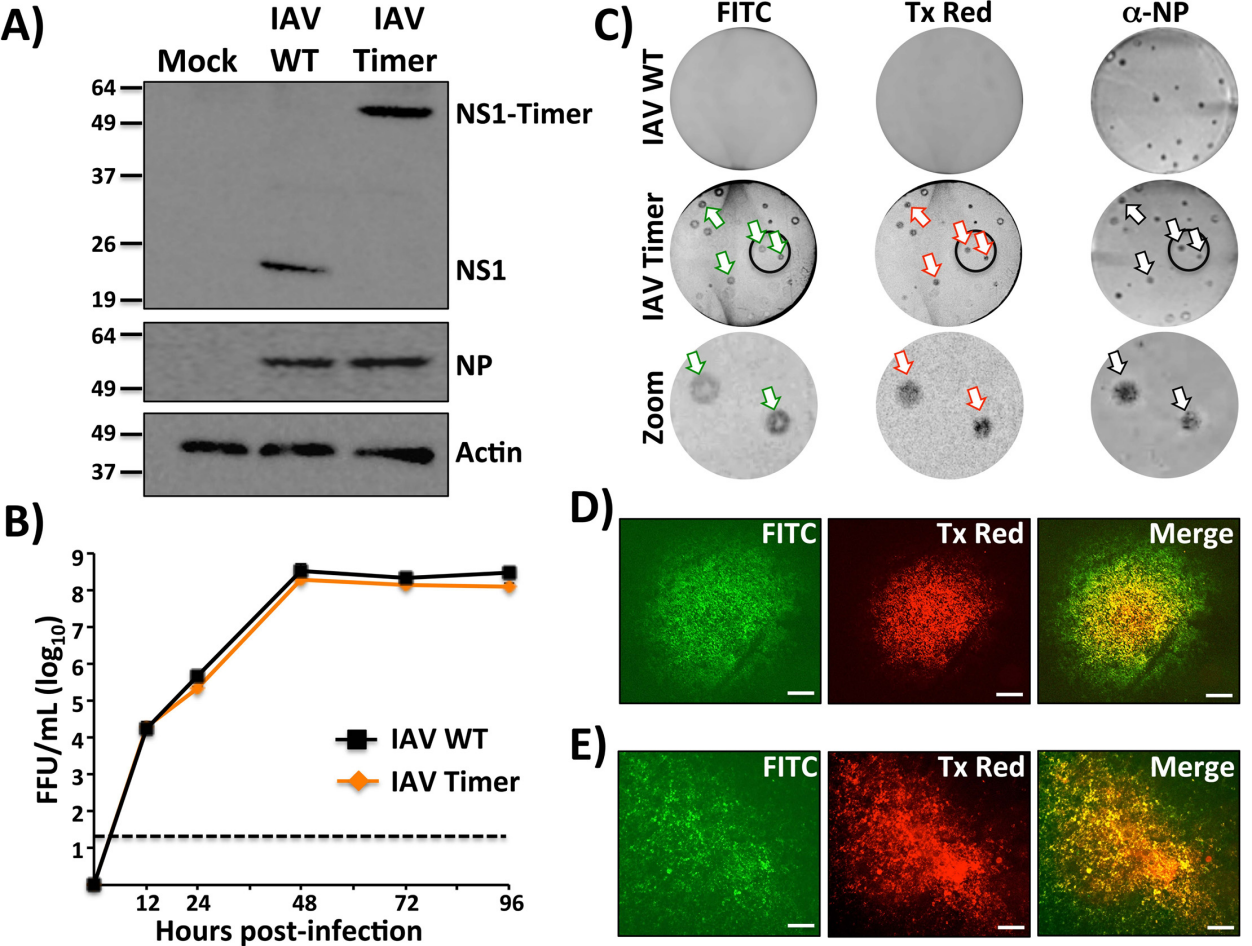
Characterization of Timer-expressing IAV. A) Analysis of protein expression by Western blot: MDCK cells were mock infected or infected (MOI 3) with WT or Timer-expressing IAVs. At 24 hpi, cell extracts were prepared and analyzed for NS1 and NP expression levels using specific antibodies. Actin was used as a loading control. Numbers indicate the size of molecular markers in kDa. B) Multicycle growth kinetics: Viral titers from TCS of MDCK cells infected (MOI 0.001) with WT and Timer-expressing IAVs at the indicated times (0, 12, 24, 48, 72 and 96 hours) were analyzed by immunofocus assay (FFU/ml). Data represent the means ± SD of the results determined from triplicates. Dotted line indicates the limit of detection (20 FFU/ml). C) Plaque phenotype: WT and Timer-expressing IAV plaques were evaluated at 3 dpi by fluorescence using filters for fluorescein isothiocyanate (FITC) or Texas Red (Tx Red) and by immunostaining using an IAV anti-NP MAb. Arrows indicate correlation between fluorescence and the immunostaining. A zoom of the areas into the black circles for IAV-Timer is showed. “Bull’s eye” plaque (D) and “comet tail” (E) assays: Monolayers of MDCK cells were infected (MOI 0.001) with IAV-Timer and covered with solid (D) or liquid (E) media. At 72 hours post-infection, infected cells were visualized using filters for FITC and Tx Red. Merged images are showed. Scale bar, 200 μm.

**Fig 3:**
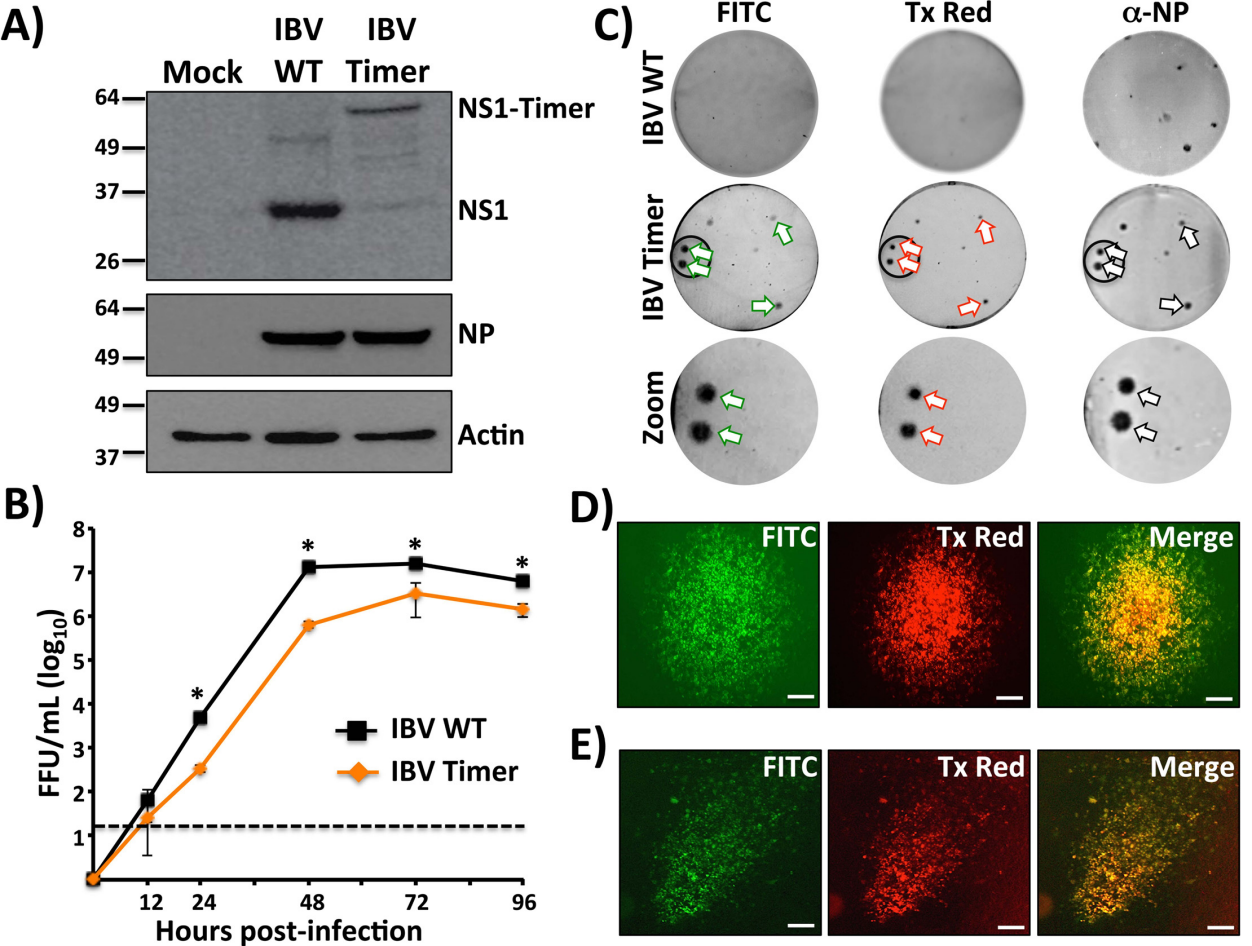
Characterization of IBV-Timer. A) Analysis of protein expression by Western blot: MDCK cells were mock infected or infected (MOI 3) with WT or Timer-expressing IBVs. At 24 hpi, cell extracts were prepared and analyzed for NS1 and NP expression levels using specific antibodies. Actin was used as a loading control. Numbers indicate the size of molecular markers in kDa. B) Multicycle growth kinetics: Viral titers from TCS of MDCK cells infected (MOI 0.001) with WT and Timer-expressing IBV were determined at the indicated times post-infection (0, 12, 24, 48, 72 and 96 hours) by immunofocus assay (FFU/ml). Data represent the means ± SD of the results determined from triplicates. Dotted line denotes the limit of detection (20 FFU/ml). *P < 0.05 using an unpaired two-tailed Student’s test. C) Plaque phenotype: Plaque sizes of WT and Timer-expressing IBVs were evaluated at 3 dpi by fluorescence using filters for fluorescein isothiocyanate (FITC) or Texas Red (Tx Red) and by immunostaining using an IBV anti-NP MAb. Arrows indicate correlation between fluorescence and the immunostaining. A zoom of the areas into the black circles for IBV-Timer is showed. “Bull’s eye” plaque (D) and “comet tail” (E) assays: Monolayers of MDCK cells were infected (MOI 0.001) with IBV-Timer and covered with solid (D) or liquid (E) media. At 72 hours post-infection, infected cells were visualized using filters for FITC and Tx Red. Merged images are showed. Scale bar, 200 μm.

To confirm that Timer was expressed as a fusion product with IAV NS1, lysates from MDCK cells mock infected or infected with IAV WT or Timer were evaluated by Western blot (Fig 2A). A band with the expected size for the NS1-Timer fusion product (~ 55 kDa) was detected in cell extracts from IAV-Timer-infected cells, while a band of lower molecular weight (~ 27 kDa) corresponding to NS1 protein was detected in cell extracts from WT-infected cells. Similarly, when Western blots were performed using cell lysates from IBV WT or Timer-infected cells, protein bands were observed for the corresponding NS1 or NS1-Timer products (~ 30 kDa and ~ 60 kDa, respectively) (Fig 3A). Specific bands of lower molecular weight were detected for IBV NS1-Timer, which could correspond to degradation products. Expression levels of NP and actin were used as infection and loading controls. NP expression levels for IAV-Timer and IBV-Timer were similar to those from WT infections, suggesting that NP expression was not affected by the NS1-Timer fusion (Figs 2A and 3A, respectively).

To evaluate the replication properties of IAV- and IBV-Timer *in vitro*, multicycle growth kinetics (MOI 0.001) were compared to WT virus in MDCK cells (Figs 2B and 3B, respectively). While IAV-Timer showed similar replication levels to IAV WT and both reached similar viral titers (Fig 2B), replication of IBV-Timer was slightly reduced as compared to IBV WT (Fig 3B). Viral plaques in cells infected with IAV (Fig 2C) or IBV (Fig 3C) Timer displayed both green and red fluorescence, and similar plaques sizes than those observed with WT IAV and IBV by immunostaining with an NP specific monoclonal antibody (Figs 2C and 3C, respectively). Importantly, plaques identified by immunostaining also showed green and red fluorescence, indicating that IAV- and IBV-Timer stably maintained a functional NS-Timer viral segment (Figs 2C and 3C, arrows). Within the same plaque, red fluorescence was more punctate and surrounded by a border of green fluorescence. This pattern is expected since the duration of viral infection and thus Timer expression, is longer for the initially infected cells in the center of the plaques (Figs 2C and 3C, Zoom). To confirm this observation, we performed a bull’s eye assay, where infected cells were incubated in solid media prior to fluorescence microscopy (Figs 2D and 3D). In the center of the plaque where the earliest infected cells reside, most cells showed red fluorescence with an outward concentric yellow/orange zone. From the red core, green infected cells predominate towards the periphery, representing newly infected cells (Figs 2D and 3D). A similar effect was observed in the “comet” assay (Figs 2E and 3E), where cells were incubated in liquid media and a characteristic comet-like appearance of viral infections was identified [43]. Here, the earliest infected cells were localized to the “head” of the comet (red fluorescence) and predominantly green cells were observed within the “tail” of the comet (Figs 2E and 3E). Like in the bull’s eye assay, yellow/orange fluorescence (merged pictures) was observed in cells in which the expected maturation phenotype of the Timer protein was occurring. Thus, by tracking fluorescence emission of Timer-expressing IAV and IBV, it is possible to evaluate, both in solid and liquid media, early (red), intermediate (red and green), and late (green) infected cells. Altogether, these assays demonstrate that IAV and IBV expressing the dynamic Timer protein can be useful for *in vitro* studies. Both fluorescent viruses have similar kinetics to their WT counterparts with the benefit of time-dependent spectral changes in fluorescence protein emission.

### Quantification of IAV- and IBV-Timer-infected MDCK cells

**Fig 4:**
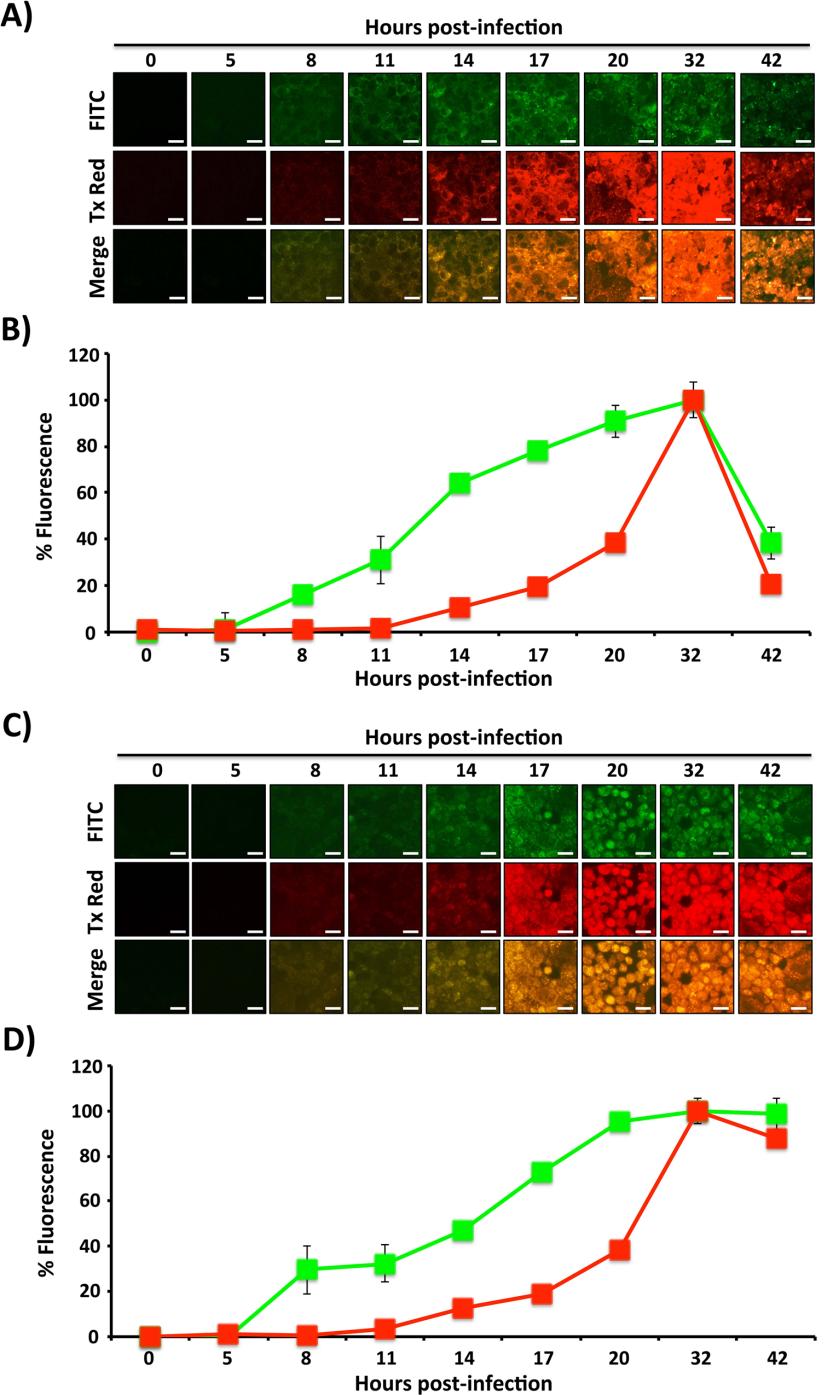
Whole population Timer fluorescence dynamics in MDCK cells infected with high MOI. MDCK cells were infected (MOI 3) with Timer-expressing IAV (A and B) or IBV (C and D) and, at the indicated times post-infection, fluorescence expression was analyzed using FITC or Tx Red filters on a fluorescence microscope (A and C). Representative images and their merge are shown. Scale bar, 25 μm. Levels of green and red fluorescence in infected cell monolayers were quantified at the same times post-infection using a fluorescence microplate reader (B and D). Data represent the means ± SD of the results determined from triplicate wells.

We next performed a time course assay to evaluate if the emission shift of Timer expression in influenza-infected cells corresponded with the duration of infection (Fig 4). To that end, MDCK cells were infected with a high MOI (3) with either IAV- or IBV-Timer viruses and then evaluated by fluorescence microscopy at different times post-infection (Fig 4A and 4C), and quantified by using a fluorescence microplate reader (Fig 4B and 4D). IAV-Timer expression was first detected at 8 hpi when exclusively green signal was emitted, with ~20% of the maximum peak of fluorescent intensity (Fig 4B). Green fluorescence expression consistently increased until ~32 hpi, but declined at 42 hpi, due to cytopathic effect (CPE) and possibly less Timer protein expression. Red fluorescence first emerged at ~11–14 hpi and continued to increase in intensity until ~32 hpi and once again declining at 42 hpi due to virus-induced CPE (Fig 4B). These readings paralleled the observations of the corresponding fluorescence images (Fig 4A). Similarly, green signal in IBV-Timer-infected cells was first observed at ~8 hpi (Fig 4D), and also reached its maximum at ~32 hpi. Red fluorescence appeared at ~11–14 hpi, and achieved its strongest intensity by ~32 hpi, following the dynamics of IBV infection. Microplate readings of IBV-Timer infected cells correlated with fluorescence images taken at the same time points post-infection (Fig 4C). These results demonstrate the measurable maturation of Timer fluorescence in a synchronized IAV or IBV infection. Both fluorescence microscopy and a microplate reader can be used to determine green versus red fluorescence emission in infected cell monolayers.

**Fig 5:**
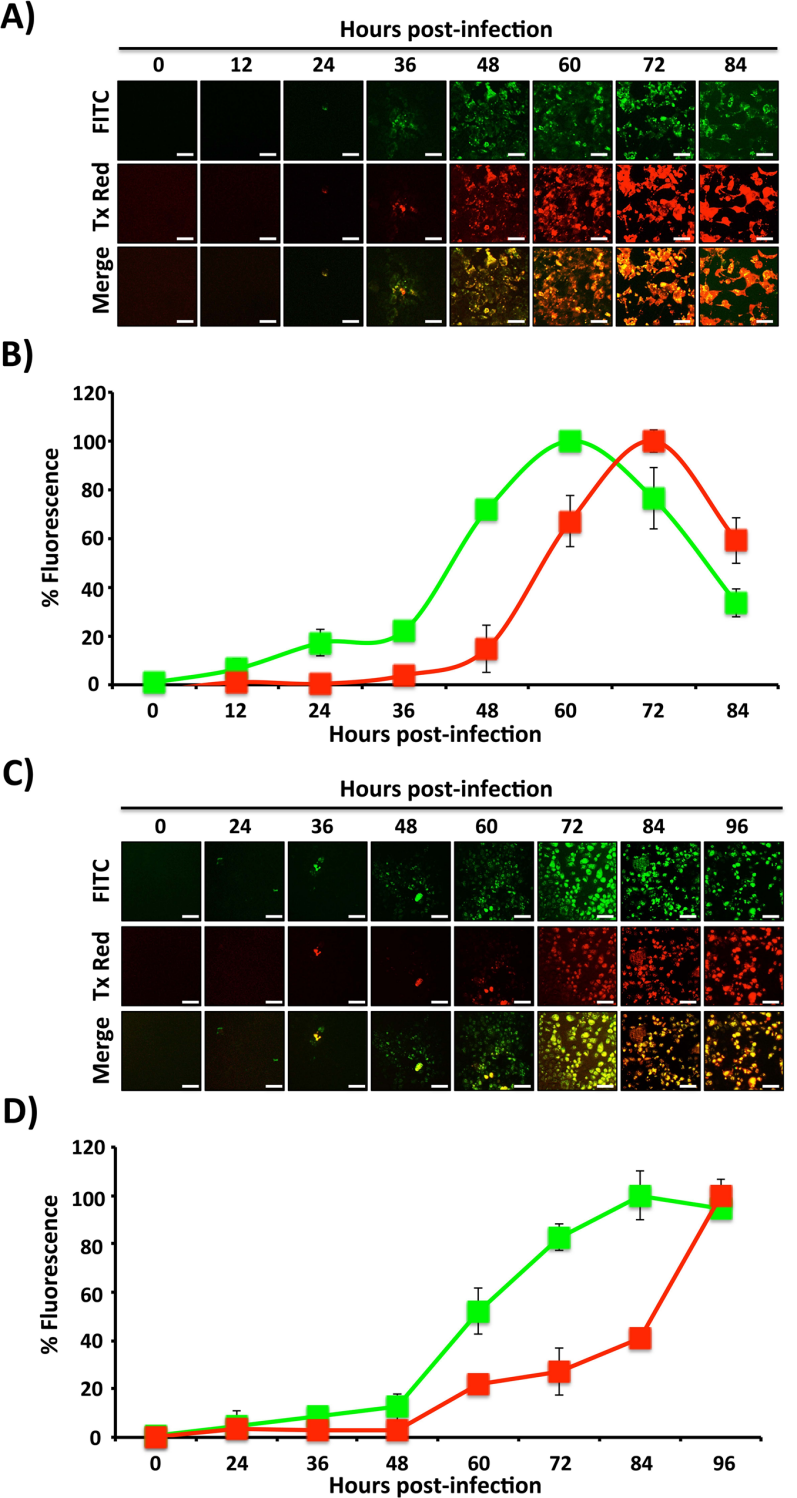
Timer-expression dynamics in MDCK cells infected with low MOI. MDCK cells were infected (MOI 0.001) with Timer-expressing IAV (A and B) or IBV (C and D) and, at the indicated times post-infection, monolayers were analyzed for Timer expression using FITC or Tx Red filters under a fluorescence microscope (A and C). Representative images and their merge are illustrated. Scale bar, 50 μm. The levels of green and red fluorescence were quantified at the same times post-infection using a fluorescent microplate reader (B and D). Data represent the means ± SDs of the results determined from triplicate wells.

We next evaluated if the Timer fluorescent protein can be used to distinguish early versus late influenza infected cells as well as the dynamics of viral infection. MDCK cells were infected using a low (0.001) MOI and green and red fluorescent Timer expression was evaluated at different time points (Fig 5). As expected, green fluorescence in both IAV- and IBV-Timer emerged before a red signal could be detected (Fig 5B and 5D, respectively). At this MOI, green fluorescence could not be observed in IAV-Timer-infected cells until ~24 hpi, showing increasing intensity with a peak maximum at ~60 hpi (Fig 5B). CPE could be observed at ~72 hpi onwards and corresponded with a decrease in green fluorescence (Fig 5A). In IBV-Timer-infected cells, green fluorescence was not observed until ~36 hpi with increased time-dependent expression, reaching a maximum value at ~84 hpi (Fig 5D). Red fluorescence was first observed at ~48 or 60 hpi for IAV- and IBV-Timer-infected cells, respectively, and reached its maximum value at ~72 and ~96 hpi for IAV-Timer and IBV-Timer, respectively (Fig 5B and 5D). IBV-Timer infected cells showed slower emergence of fluorescence than IAV-Timer-infected cells, corresponding to IBV’s slower growth kinetics (compared Figs 2B and 3B). Fluorescence images (Fig 5A and 5C) recapitulate values detected by the fluorescent microplate reader. These data suggest that Timer-expressing recombinant IAV and IBV viruses can be used to track and quantify the dynamics of influenza A and B viral infections in tissue culture.

### Flow cytometry quantification of IAV-Timer infection

**Fig 6:**
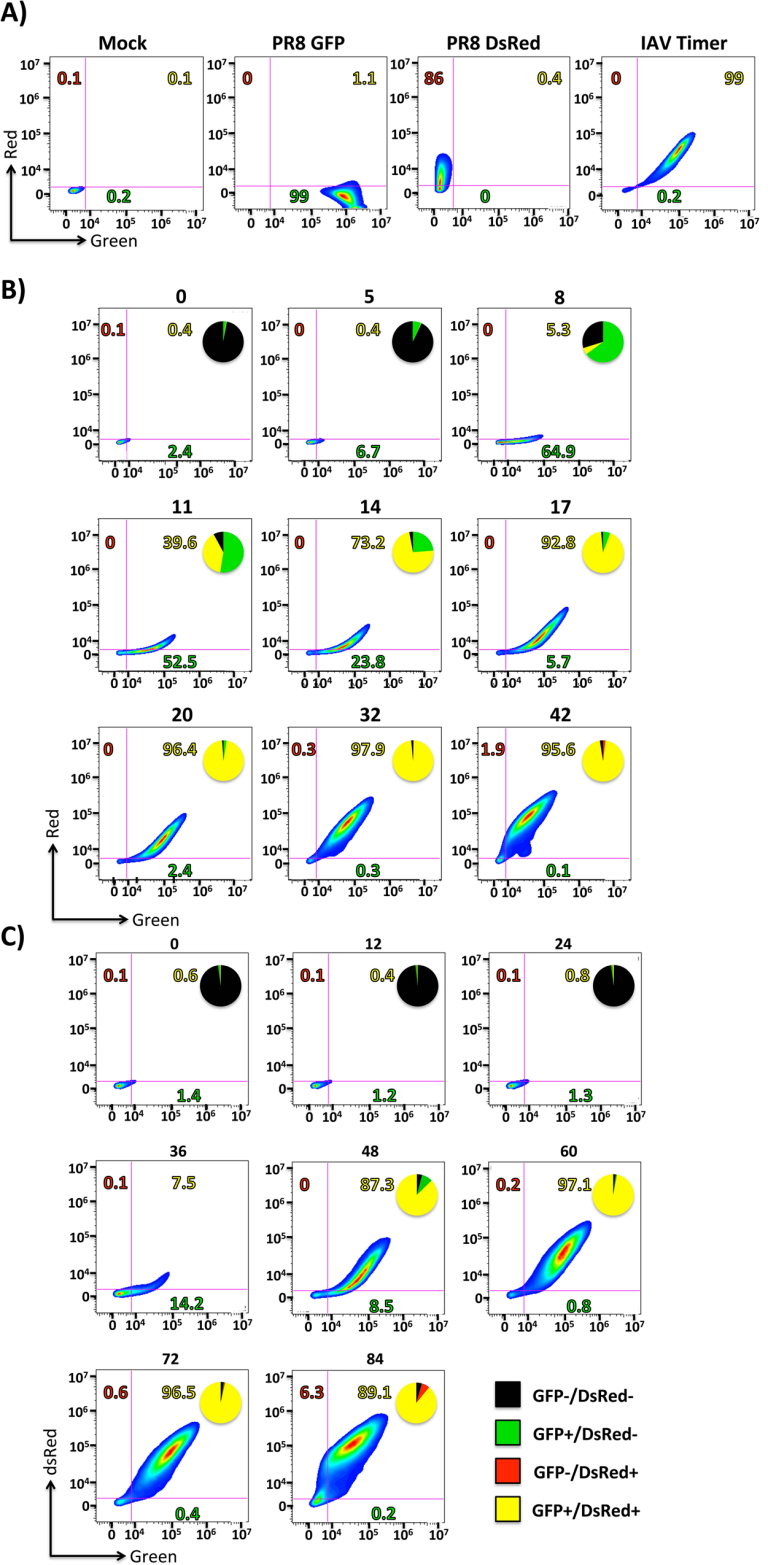
Flow cytometric analysis of cells infected with Timer-expressing IAV. A) Dual-fluorescence of Timer-infected cells: MDCK cells were either mock infected or infected (MOI 3) with GFP-, DsRed- or Timer-expressing IAV. At 24 hpi, infected cell suspensions were analyzed and quantified for fluorescence expression using flow cytometry. B and C) Analysis of Timer expression from IAV-infected cells: MDCK cells were infected at high (3) (B) or low (0.001) (C) MOI and analyzed for fluorescence expression at the indicated times post-infection. Gates were set on mock-infected cells. Pie charts within plots indicate the percentage of each population GFP-/DsRed- (black), GFP+/DsRed- (green), GFP-/DsRed+ (red) and GFP+/DsRed+ (yellow).

To measure the duration of influenza infection on a cellular level, MDCK cells infected with Timer-expressing IAV were evaluated by flow cytometry (Fig 6). Cells mock infected or infected with PR8 viruses expressing GFP or DsRed at high MOI (3) were used as controls (Fig 6A). At 24 hours post-infection, nearly 100% of IAV-Timer-infected cells displayed both green and red fluorescence. In contrast and as expected, PR8 GFP and PR8 DsRed infection controls displayed only green or red fluorescence, respectively (Fig 6A). This observation further confirmed the dual-emission capability of Timer during IAV infection.

To determine the rate of NS1-Timer spectral shift, MDCK cells were infected at a high (3) MOI to achieve a synchronized infection. Similar to the data at the population level (Fig 4), flow cytometric analysis shows individual infected cells first emit green fluorescence, which was clearly detected at 8 hpi (~65% cell expressing green fluorescence versus ~5% of cells expressing both green and red fluorescence). By 11 hpi, ~50% of the cells showed exclusive green fluorescence while ~40% showed both green and red fluorescence. Between 8 and 32 hpi, the percentage of green fluorescent cells decreased from their maximum of ~65% (8 hpi) to ~0% (32 hpi) (Fig 6B). Over time, the emission spectra of infected cells shifted as a population of double-positive fluorescent cells, starting with ~5% at 8 hpi, reaching ~92% at 17 hpi; and peaking with ~98% at 32 hpi (Fig 6B). These results mirror those obtained with the microplate reader that shows each fluorescent signal reached its respective higher value at 32 hpi (Fig 4B). Finally, at 42 hpi, most infected cells increased in red fluorescence intensity and decreased in green, as indicated by points shifting left and up on the *x*- and *y*-axes (Fig 6C).

We also measured the fluorescent signal produced by IAV-Timer-infected cells at low (0.001) MOI to examine virus spread into uninfected cells (Fig 6C). Infected cells did not exhibit fluorescent signal until ~36 hpi, when green fluorescence was first detected in ~14% of cells. Timer signal at this point had already begun to shift towards red in ~7.5% of the infected cells. By 48 hpi, ~87% of infected cells expressed both green and red fluorescence; and at 60 hpi, nearly all (~97%) of the cells emitted green and red fluorescence. These percentages were maintained until 84 hpi, when the dual-fluorescence decreased (~89.1%) and a population of exclusively red fluorescent cells (~6.3%) was detected. We were not able to visualize fluorescence expression at later times post-infection because of virus-induced CPE (data not shown). Thus, flow cytometry analysis of IAV-Timer-infected cells at high and low MOI shows stratification of the fluorescence emission (from green to red), which allows for separation between early, intermediate and late times post-infection based on GFP, GFP and DsRed, and DsRed expression, respectively.

### Kinetics of Timer expression and IAV replication in mice lungs

**Fig 7:**
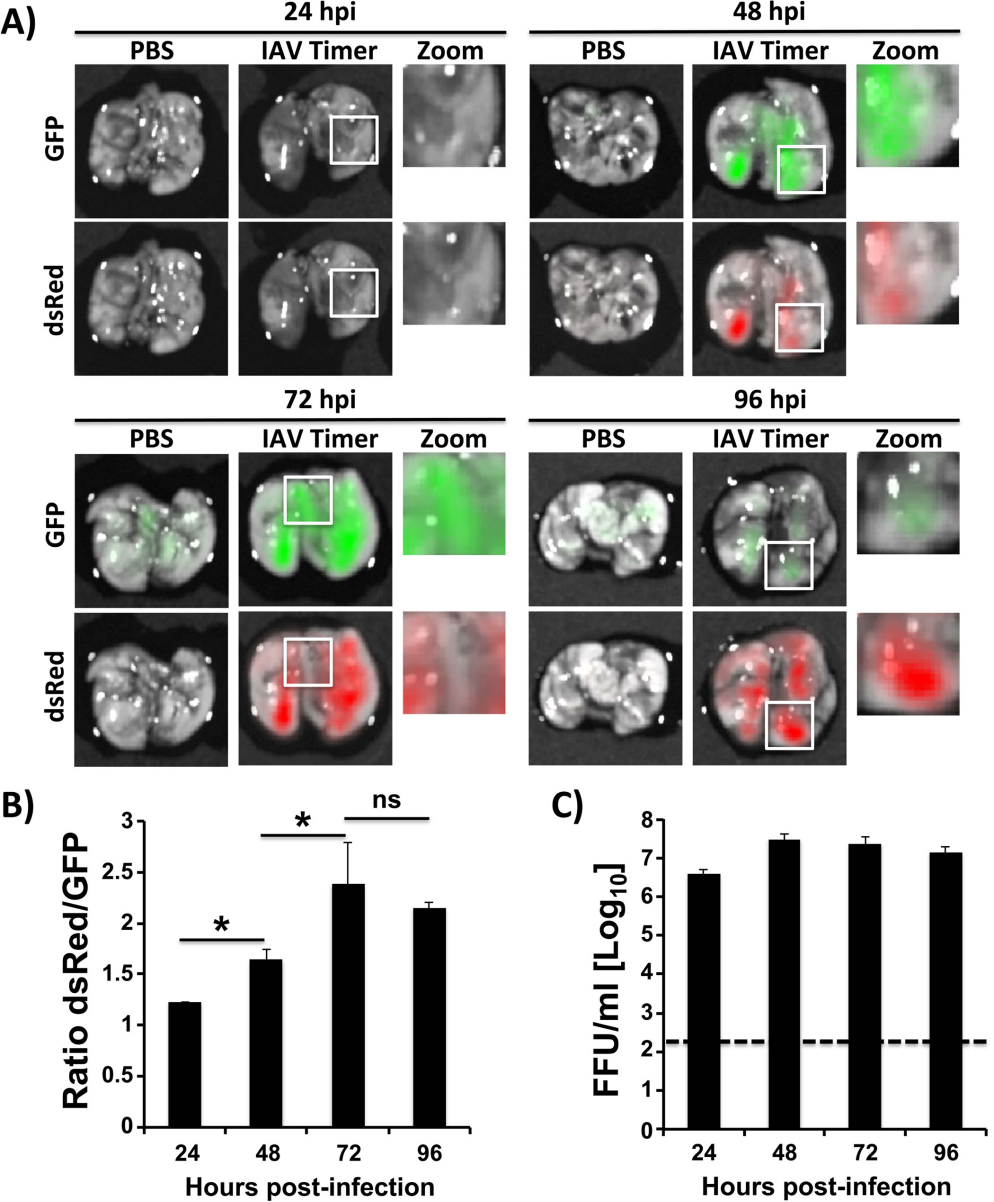
Kinetics of IAV-Timer infection in mouse lungs. Female 6-to-8-week-old C57BL/6 mice (n = 3) were inoculated intranasally with PBS or with 10^5^ PFU of IAV-Timer. At 24, 48, 72 and 96 hpi, mice lungs were excised to evaluate and quantify fluorescence (A and B) and production of infectious virus (C). A and B) Fluorescence imaging of infected lungs: Lungs from mock infected (PBS) and IAV-Timer-infected mice were harvested and analyzed by IVIS (A). Representative fluorescence images are shown. The radiant efficiency fold induction from individual mice using DsRed or GFP filters was normalized to mock infected mice. The average ratio of DsRed/GFP expression was determined at each time point (B). Columns represent mean +/- SD. Statistical significance was calculated using two-tailed Student’s *t* test. * indicates P values < 0.05. C) Viral lung titers. Lung homogenates were used to quantify presence of virus by immunofocus assay (FFU/ml). Bars represent the mean +/-SD. Dotted line denotes the limit of detection (200 FFU/ml).

To assess whether IAV-Timer could be used to study the dynamics of IAV replication *in vivo*, the green to red fluorescence shift was evaluated in lungs excised from mice infected with IAV-Timer (Fig 7). To this end, lungs from mice inoculated with PBS or 10^5^ PFU of IAV-Timer were surgically removed at different stages post-infection (24, 48, 72 and 96 hpi). Lung fluorescence expression was evaluated *ex vivo* by using an IVIS Spectrum multispectral imaging instrument evaluating GFP and DsRed fluorescence (Fig 7A), and the ratio of the mean radiant efficiency of red to green fluorescence was calculated (Fig 7B). Induction of fluorescence signal was normalized to mock-infected animals. Green and red fluorescence was observed at 48 through 96 hpi (Fig 7A). The intensity of green fluorescence was higher at 48–72 hpi and decreased at 96 hpi, while the intensity of red fluorescence was lower at 48–72 hpi and increased at 96 hpi (Fig 7A). Importantly, IAV spread could be examined by characterizing pulmonary areas that were predominantly green (white squares at 48–72 hpi), a combination of green and red (white squares at 72 hpi) or predominantly red (white squares at 96 hpi). In agreement with *in vitro* data, areas in the lungs of infected mice correspond with early (green), intermediate (green and red) or late (red) infected cells. The red:green fluorescence ratio throughout the lungs was calculated, showing that fluorescence shifted from green to red in a time-dependent manner (Fig 7B). Importantly, fluorescence expression correlated with viral titers in the lungs from infected mice (Fig 7C). These findings indicate that IAV-Timer can be used to track viral infection in mouse lungs and to determine early (green), intermediate (green and red) and late (red) infected cells during the course of IAV infection *in vivo*.

### Genetic stability of Timer-expressing IAV and IBV

To analyze the genetic stability of the reporter Timer-expressing influenza viruses, three independent clones were passaged five times in MDCK cells and the percentage of reporter-expressing viruses was determined by plaque assay and fluorescent microscopy. As shown in Table 1, both IAV- and IBV-Timer retained 90–100% reporter gene expression at least during the first three subsequent serial passages. Notably, after five consecutive passages, more than 50% of the IAB or IBV retained reporter gene expression.

**Table 1:** Genetic stability of IAV- and IBV-Timer.

	IAV-Timer			IBV-Timer		
Passage	Clone 1	Clone 2	Clone 3	Clone 1	Clone 2	Clone 3
**P1**	100	100	100	100	100	100
**P2**	100	100	100	100	100	100
**P3**	93	96	90	91	93	98
**P4**	84.4	86	77.5	85	75	87
**P5**	63	75	50	73	52.5	58

## Discussion

IAV expressing fluorescent proteins have proven useful to measure viral localization and replication [18, 44] or to evaluate neutralizing antibodies (NAbs) and antivirals [13, 45] *in vitro*. Likewise, fluorescent IAV provides a means to monitor viral infection in real time, which can be used to study viral spread and evaluate antivirals *in vivo* [13, 17, 20, 21, 33, 34]. Here, we describe the generation and characterization of replication-competent IAV and IBV expressing a fluorescent dynamic Timer protein fused to NS1 (Fig 1). Importantly, Timer-expressing IAV and IBV have similar viral titers with slightly reduced replication kinetics as compared with their WT counterparts (Figs 2 and 3).

Timer offers a way to identify and determine the approximate chronology of infected cells (Figs 2D and 3D; and Figs 2E and 3E) because the fluorescent protein has the unique characteristic of shifting from green (early) to red (late) fluorescence over time (Fig 4A and 4C; and Fig 5A and 5C) [38]. This fluorescence shift can be also quantified *in vitro* using fluorescence microplate readers (Fig 4B and 4D; and Fig 5B and 5D), or flow cytometry (Fig 5C and 5D). Importantly, early, intermediate, and late infected cells can be easily identified and could be isolated by fluorescence-activated cell sorting (FACS). Therefore, both influenza Timer viruses could serve to model virus replication and infection in real-time. This represents an important advantage over previous static-fluorescent protein influenza viruses that cannot be used to determine the duration of cellular infection [13, 17–25]. On the contrary, IAV- and IBV-Timer could be used to identify early and late infections based on the spectral phenotype. Early-infected cells showed solely green emission while late infected cells displayed a combination of both green and red forms of Timer protein. This is because new Timer protein is continually synthesized during the course of infection. We would like to speculate that this dynamic fluorescence expression by recombinant influenza viruses could be used to dissect host responses to viral infection at early versus late times post-infection.

The within-host dissemination of IAV remains poorly characterized. When IAV-Timer infection was monitored *ex vivo*, the fluorescence profile in the lungs of infected mice recapitulated the *in vitro* data (Fig 7). Green fluorescence was detected earlier than red fluorescence and decreased over time in infected mouse lungs, coinciding with an increase of red fluorescence. Moreover, the pulmonary images revealed that areas with green fluorescence, but not red, can be observed and vice versa. These fluorescent pulmonary regions most likely correspond to early and late infected cells, suggesting that virus dissemination can be tracked *in vivo*.

IAV- and IBV-Timer could be of particular use to evaluate the changes in host gene expression at different stages of viral infection because we are able to identify cells with a recent infection (green) and cells with a sustained infection (red). Early (green), intermediate (green and red), and late (red) infected cells could be sorted by FACS and have their gene expression profiles analyzed to evaluate host responses at different times post-infection. Further analysis of the turnover of Timer protein can lead to identifying “coordinates” that correspond to precise infection timing. This could lead to the identification and characterization of genes expressed during varying stages of viral infection and the timing required for the activation of host restriction factors induced by type-I interferon, such as MxA [46]. Infection of mice with IAV-Timer could also be further implemented to determine the rate of virus dissemination in tissue, using the dual-fluorescent nature of this reporter fluorescent protein, by tracking red fluorescent cells (oldest infected) and green fluorescent cells (newly infected). A number of studies incorporating static-fluorescent protein influenza viruses have assessed the transcriptomics and proteomics profiles of influenza virus-infected mice [23, 47, 48]. Recently, Fukuyama *et al*. used replication-competent viruses harboring fluorescent proteins for differential gene expression studies in virus antigen-positive and virus antigen-negative live cells in the lungs of infected mice [23]. However, these studies were not able to distinguish the profiles of early and late infected cells. Timer-expressing IAV or IBV could be used to sort cells based on their green, green and red, or red fluorescence expression to explore the time-dependent expression of restriction and virulence factors. In conclusion, IAV- and IBV-Timer in combination with advanced technologies [49] to visualize and quantify fluorescent proteins represent a powerful, versatile and novel approach to elucidate the mechanisms of influenza virus infection, dissemination and pathogenicity *in vitro* and *in vivo*. Likewise, Timer-expressing IAV and IBV can be used to identify and sort cells at different stages of infection, opening up the possibility of characterizing host factors regulated at different times post-infection.

## References

[1] CDC. Deaths: Final Data for 2013. 2013 February 6. Report No.

[2] WHO. Influenza (Seasonal) Fact Sheet N211 http://www.who.int/mediacentre/factsheets/fs211/en/2014 [November 2]. Available from: http://www.who.int/mediacentre/factsheets/fs211/en/.

[3] Simonsen L, Clarke MJ, Williamson GD, Stroup DF, Arden NH, Schonberger LB. The impact of influenza epidemics on mortality: introducing a severity index. Am J Public Health. 1997;87(12):1944–50. Epub 1998/02/07. ; PubMed Central PMCID: PMCPmc1381234. 9431281 10.2105/ajph.87.12.1944 PMC1381234

[4] CDC. Updated CDC Estimates of 2009 H1N1 Influenza Cases, Hospitalizations and Deaths in the United States, April 2009–April 10, 2010 2010 [updated May 14; cited 2013]. Available from: http://www.cdc.gov/h1n1flu/estimates_2009_h1n1.htm.

[5] Dawood FS, Iuliano AD, Reed C, Meltzer MI, Shay DK, Cheng P-Y, et al Estimated global mortality associated with the first 12 months of 2009 pandemic influenza A H1N1 virus circulation: a modelling study. The Lancet Infectious Diseases. 2012;12(9):687–95. 10.1016/S1473-3099(12)70121-4 22738893

[6] Influenza-associated pediatric deaths—United States, September 2010-August 2011. MMWR Morb Mortal Wkly Rep. 2011;60(36):1233–8. Epub 2011/09/16. . 21918492

[7] Palese P, Shaw M. Orthomyxoviridae In: Knipe D, Howley P, Griffin D, Lamb R, Martin M, Roizman B, et al, editors. Fields Virology. I. 6 ed. Philadelphia: Lippincott Williams and Wilkins; 2013.

[8] Compans RW, Content J, Duesberg PH. Structure of the Ribonucleoprotein of Influenza Virus. Journal of Virology. 1972;10(4):795–800. . 4117350 10.1128/jvi.10.4.795-800.1972 PMC356535

[9] Neumann G, Watanabe T, Ito H, Watanabe S, Goto H, Gao P, et al Generation of influenza A viruses entirely from cloned cDNAs. Proc Natl Acad Sci U S A. 1999;96(16):9345–50. Epub 1999/08/04. ; PubMed Central PMCID: PMCPmc17785. 10430945 10.1073/pnas.96.16.9345 PMC17785

[10] Fodor E, Devenish L, Engelhardt OG, Palese P, Brownlee GG, Garcia-Sastre A. Rescue of influenza A virus from recombinant DNA. J Virol. 1999;73(11):9679–82. Epub 1999/10/09. ; PubMed Central PMCID: PMCPmc113010. 10516084 10.1128/jvi.73.11.9679-9682.1999 PMC113010

[11] Martinez-Sobrido L, Garcia-Sastre A. Generation of recombinant influenza virus from plasmid DNA. J Vis Exp. 2010;(42). Epub 2010/08/24. 10.3791/2057 ; PubMed Central PMCID: PMCPmc3156010. 20729804 PMC3156010

[12] Subbarao K, Katz JM. Influenza vaccines generated by reverse genetics. Curr Top Microbiol Immunol. 2004;283:313–42. Epub 2004/08/10. . 15298174 10.1007/978-3-662-06099-5_9

[13] Nogales A, Baker SF, Martinez-Sobrido L. Replication-competent influenza A viruses expressing a red fluorescent protein. Virology. 2014;476c:206–16. Epub 2015/01/02. 10.1016/j.virol.2014.12.006 . 25553516 PMC4323957

[14] Ozawa M, Kawaoka Y. Taming influenza viruses. Virus Res. 2011;162(1–2):8–11. Epub 2011/10/05. 10.1016/j.virusres.2011.09.035 ; PubMed Central PMCID: PMCPmc3223391. 21968297 PMC3223391

[15] Baker SF, Nogales A, Finch C, Tuffy KM, Domm W, Perez DR, et al Influenza A and B virus intertypic reassortment through compatible viral packaging signals. J Virol. 2014;88(18):10778–91. Epub 2014/07/11. 10.1128/jvi.01440-14 ; PubMed Central PMCID: PMCPmc4178878. 25008914 PMC4178878

[16] Roberts KL, Manicassamy B, Lamb RA. Influenza A virus uses intercellular connections to spread to neighboring cells. J Virol. 2015;89(3):1537–49. Epub 2014/11/28. 10.1128/jvi.03306-14 ; PubMed Central PMCID: PMCPmc4300760. 25428869 PMC4300760

[17] Eckert N, Wrensch F, Gartner S, Palanisamy N, Goedecke U, Jager N, et al Influenza A virus encoding secreted Gaussia luciferase as useful tool to analyze viral replication and its inhibition by antiviral compounds and cellular proteins. PLoS One. 2014;9(5):e97695 Epub 2014/05/21. 10.1371/journal.pone.0097695 ; PubMed Central PMCID: PMCPmc4026478. 24842154 PMC4026478

[18] Pan W, Dong Z, Li F, Meng W, Feng L, Niu X, et al Visualizing influenza virus infection in living mice. Nat Commun. 2013;4:2369 Epub 2013/09/12. 10.1038/ncomms3369 ; PubMed Central PMCID: PMCPmc3778511. 24022374 PMC3778511

[19] Tran V, Moser LA, Poole DS, Mehle A. Highly sensitive real-time in vivo imaging of an influenza reporter virus reveals dynamics of replication and spread. J Virol. 2013;87(24):13321–9. Epub 2013/10/04. 10.1128/jvi.02381-13 ; PubMed Central PMCID: PMCPmc3838222. 24089552 PMC3838222

[20] Kittel C, Sereinig S, Ferko B, Stasakova J, Romanova J, Wolkerstorfer A, et al Rescue of influenza virus expressing GFP from the NS1 reading frame. Virology. 2004;324(1):67–73. Epub 2004/06/09. 10.1016/j.virol.2004.03.035 . 15183054

[21] Manicassamy B, Manicassamy S, Belicha-Villanueva A, Pisanelli G, Pulendran B, Garcia-Sastre A. Analysis of in vivo dynamics of influenza virus infection in mice using a GFP reporter virus. Proc Natl Acad Sci U S A. 2010;107(25):11531–6. Epub 2010/06/11. 10.1073/pnas.0914994107 ; PubMed Central PMCID: PMCPmc2895123. 20534532 PMC2895123

[22] Perez JT, García-Sastre A, Manicassamy B. Insertion of a GFP Reporter Gene in Influenza Virus Current Protocols in Microbiology: John Wiley & Sons, Inc.; 2013. 10.1002/9780471729259.mc15g04s29 PMC3878617 23686828

[23] Fukuyama S, Katsura H, Zhao D, Ozawa M, Ando T, Shoemaker JE, et al Multi-spectral fluorescent reporter influenza viruses (Color-flu) as powerful tools for in vivo studies. Nat Commun. 2015;6:6600 10.1038/ncomms7600 25807527 PMC4389232

[24] Fiege JK, Langlois RA. Investigating influenza A virus infection: tools to track infection and limit tropism. J Virol. 2015;89(12):6167–70. 10.1128/JVI.00462-15 25855737 PMC4474293

[25] Reuther P, Gopfert K, Dudek AH, Heiner M, Herold S, Schwemmle M. Generation of a variety of stable Influenza A reporter viruses by genetic engineering of the NS gene segment. Sci Rep. 2015;5:11346 10.1038/srep11346 26068081 PMC4464305

[26] Fulton BO, Palese P, Heaton NS. Replication-Competent Influenza B Reporter Viruses as Tools for Screening Antivirals and Antibodies. J Virol. 2015;89(23):12226–31. 10.1128/JVI.02164-15 . 26401044 PMC4645317

[27] Heaton NS, Leyva-Grado VH, Tan GS, Eggink D, Hai R, Palese P. In vivo bioluminescent imaging of influenza a virus infection and characterization of novel cross-protective monoclonal antibodies. J Virol. 2013;87(15):8272–81. 10.1128/JVI.00969-13 23698304 PMC3719835

[28] Karlsson EA, Meliopoulos VA, Savage C, Livingston B, Mehle A, Schultz-Cherry S. Visualizing real-time influenza virus infection, transmission and protection in ferrets. Nat Commun. 2015;6:6378 10.1038/ncomms7378 25744559 PMC4366512

[29] Spronken MI, Short KR, Herfst S, Bestebroer TM, Vaes VP, van der Hoeven B, et al Optimisations and Challenges Involved in the Creation of Various Bioluminescent and Fluorescent Influenza A Virus Strains for In Vitro and In Vivo Applications. PLoS One. 2015;10(8):e0133888 10.1371/journal.pone.0133888 26241861 PMC4524686

[30] Sutton TC, Obadan A, Lavigne J, Chen H, Li W, Perez DR. Genome rearrangement of influenza virus for anti-viral drug screening. Virus Res. 2014;189:14–23. 10.1016/j.virusres.2014.05.003 24833536 PMC4134972

[31] Yan D, Weisshaar M, Lamb K, Chung HK, Lin MZ, Plemper RK. Replication-Competent Influenza Virus and Respiratory Syncytial Virus Luciferase Reporter Strains Engineered for Co-Infections Identify Antiviral Compounds in Combination Screens. Biochemistry. 2015;54(36):5589–604. 10.1021/acs.biochem.5b00623 . 26307636 PMC4719150

[32] Zhao D, Fukuyama S, Yamada S, Lopes TJ, Maemura T, Katsura H, et al Molecular Determinants of Virulence and Stability of a Reporter-Expressing H5N1 Influenza A Virus. J Virol. 2015;89(22):11337–46. 10.1128/JVI.01886-15 . 26339046 PMC4645634

[33] Pena L, Sutton T, Chockalingam A, Kumar S, Angel M, Shao H, et al Influenza viruses with rearranged genomes as live-attenuated vaccines. J Virol. 2013;87(9):5118–27. Epub 2013/03/02. 10.1128/jvi.02490-12 ; PubMed Central PMCID: PMCPmc3624320. 23449800 PMC3624320

[34] Vieira Machado A, Naffakh N, Gerbaud S, van der Werf S, Escriou N. Recombinant influenza A viruses harboring optimized dicistronic NA segment with an extended native 5' terminal sequence: induction of heterospecific B and T cell responses in mice. Virology. 2006;345(1):73–87. Epub 2005/11/08. 10.1016/j.virol.2005.09.050 . 16271378

[35] Hale BG, Randall RE, Ortin J, Jackson D. The multifunctional NS1 protein of influenza A viruses. J Gen Virol. 2008;89(Pt 10):2359–76. Epub 2008/09/18. 10.1099/vir.0.2008/004606-0 . 18796704

[36] Dauber B, Heins G, Wolff T. The Influenza B Virus Nonstructural NS1 Protein Is Essential for Efficient Viral Growth and Antagonizes Beta Interferon Induction. Journal of Virology. 2004;78(4):1865–72. 10.1128/JVI.78.4.1865-1872.2004 PMC369500. 14747551 PMC369500

[37] Krug RM, Etkind PR. Cytoplasmic and nuclear virus-specific proteins in influenza virus-infected MDCK cells. Virology. 1973;56(1):334–48. Epub 1973/11/01. . 4795673 10.1016/0042-6822(73)90310-3

[38] Terskikh A, Fradkov A, Ermakova G, Zaraisky A, Tan P, Kajava AV, et al "Fluorescent Timer": Protein That Changes Color with Time. Science. 2000;290(5496):1585–8. 10.1126/science.290.5496.1585 11090358

[39] Quinlivan M, Zamarin D, Garcia-Sastre A, Cullinane A, Chambers T, Palese P. Attenuation of equine influenza viruses through truncations of the NS1 protein. J Virol. 2005;79(13):8431–9. Epub 2005/06/16. 10.1128/jvi.79.13.8431-8439.2005 ; PubMed Central PMCID: PMCPmc1143746. 15956587 PMC1143746

[40] Baker SF, Guo H, Albrecht RA, Garcia-Sastre A, Topham DJ, Martinez-Sobrido L. Protection against lethal influenza with a viral mimic. J Virol. 2013;87(15):8591–605. 10.1128/JVI.01081-13 23720727 PMC3719819

[41] O'Neill RE, Talon J, Palese P. The influenza virus NEP (NS2 protein) mediates the nuclear export of viral ribonucleoproteins. EMBO J. 1998;17(1):288–96. 10.1093/emboj/17.1.288 9427762 PMC1170379

[42] Hai R, Martinez-Sobrido L, Fraser KA, Ayllon J, Garcia-Sastre A, Palese P. Influenza B virus NS1-truncated mutants: live-attenuated vaccine approach. J Virol. 2008;82(21):10580–90. Epub 2008/09/05. 10.1128/jvi.01213-08 ; PubMed Central PMCID: PMCPmc2573209. 18768976 PMC2573209

[43] Gambaryan AS, Matrosovich MN, Bender CA, Kilbourne ED. Differences in the biological phenotype of low-yielding (L) and high-yielding (H) variants of swine influenza virus A/NJ/11/76 are associated with their different receptor-binding activity. Virology. 1998;247(2):223–31. 10.1006/viro.1998.9274 . 9705915

[44] Avilov SV, Moisy D, Munier S, Schraidt O, Naffakh N, Cusack S. Replication-competent influenza A virus that encodes a split-green fluorescent protein-tagged PB2 polymerase subunit allows live-cell imaging of the virus life cycle. J Virol. 2012;86(3):1433–48. Epub 2011/11/25. 10.1128/jvi.05820-11 ; PubMed Central PMCID: PMCPmc3264389. 22114331 PMC3264389

[45] Gabor KA, Goody MF, Mowel WK, Breitbach ME, Gratacap RL, Witten PE, et al Influenza A virus infection in zebrafish recapitulates mammalian infection and sensitivity to anti-influenza drug treatment. Disease Models & Mechanisms. 2014;7(11):1227–37. 10.1242/dmm.014746 25190709 PMC4213727

[46] Sadler AJ, Williams BR. Dynamiting viruses with MxA. Immunity. 2011;35(4):491–3. Epub 2011/11/01. 10.1016/j.immuni.2011.10.005 . 22035841

[47] Go JT, Belisle SE, Tchitchek N, Tumpey TM, Ma W, Richt JA, et al 2009 pandemic H1N1 influenza virus elicits similar clinical course but differential host transcriptional response in mouse, macaque, and swine infection models. BMC Genomics. 2012;13:627 10.1186/1471-2164-13-627 23153050 PMC3532173

[48] Zhao D, Liang L, Li Y, Liu L, Guan Y, Jiang Y, et al Proteomic analysis of the lungs of mice infected with different pathotypes of H5N1 avian influenza viruses. Proteomics. 2012;12(12):1970–82. 10.1002/pmic.201100619 . 22623221

[49] Lim K, Hyun YM, Lambert-Emo K, Capece T, Bae S, Miller R, et al Neutrophil trails guide influenza-specific CD8(+) T cells in the airways. Science. 2015;349(6252):aaa4352 10.1126/science.aaa4352 . 26339033 PMC4809646

